# The Role of Relationship Quality for Solitude Experiences during the Pandemic

**DOI:** 10.1093/geroni/igab046.3648

**Published:** 2021-12-17

**Authors:** Yoonseok Choi, Theresa Pauly, Elizabeth Zambrano Garza, Tiana Broen, Denis Gerstorf, Christiane Hoppmann

**Affiliations:** 1 University of British Columbia, Vancouver, British Columbia, Canada; 2 University of Zurich, University of Zurich, Zurich, Switzerland; 3 Humboldt University of Berlin, Humboldt University of Berlin, Brandenburg, Germany; 4 University of British Columbia, University of British Columbia, British Columbia, Canada

## Abstract

As time spent at home has significantly increased during the pandemic, reports of household conflict has also risen among people living with others (Usher et al., 2020). One solution to alleviate the potential stress of increased time with others could be carving out time to oneself. The present study investigated how living conditions (e.g., with others vs. alone) are associated with everyday desire for solitude and whether daily solitude experience comes with improved daily emotional well-being in people living with others. Furthermore, it also explored whether relationship quality is associated with solitude experience in a similar manner as living conditions. To do so, we used repeated daily life assessments from a lifespan sample (N = 215; M age = 38.3 years, SD age = 17.5; 78 % female) collected during the early pandemic (April to August 2020). Findings indicate that neither living conditions nor relationship quality were directly associated with daily desire for solitude, but higher relationship well-being was related to low preference for solitude when measured as an individual trait. In addition, relationship quality significantly moderated everyday solitude–affect links: higher relationship quality was related to reduced negative affect during solitude, and conflict was related to increased positive and decreased negative affect on solitude as compared to non-solitude days. The results imply that it is the subjective experience of relationships rather than objective living conditions that shape daily affective quality during solitude.

